# Supplementary material for: Silencing the Tlr4 Gene Alleviates Methamphetamine-Induced Hepatotoxicity by Inhibiting Lipopolysaccharide-Mediated Inflammation in Mice
Source: Int J Mol Sci. 2022 Jun 18;23(12):6810. doi: 10.3390/ijms23126810 (PMC9224410; doi:10.3390/ijms23126810)
Supplement: Supplementary file 1 [file ijms-23-06810-s001.zip › ijms-1737131-supplementary.pdf]

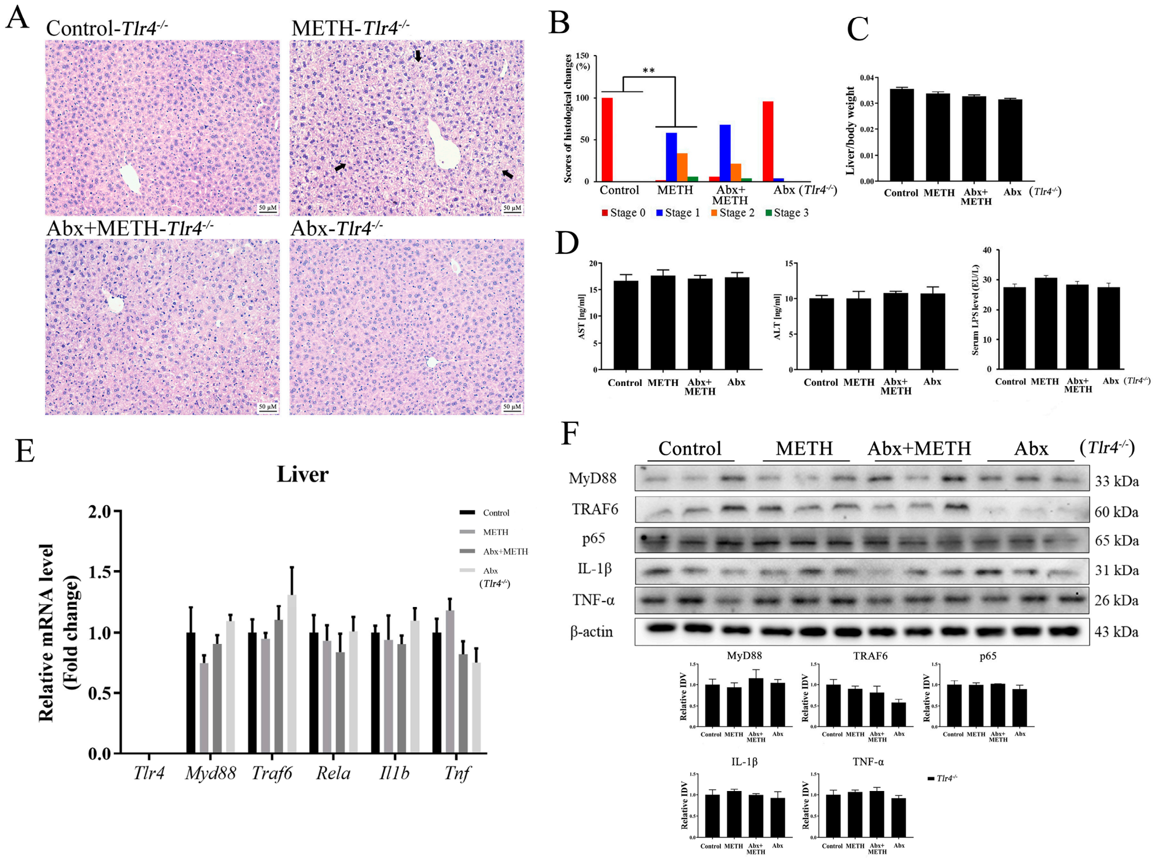


**Supplementary Figure S1** The effect of METH treatment on inflammation in the livers of *Tlr4*$^{-/-}$ mice. (A) Histopathological observation. (B) The scores of histological changes. (C) The relative liver weights. (D) Effect of METH on serum AST, ALT, and LPS levels. (E) The mRNA expression of *Tlr4*, *Myd88*, *Traf6*, *Rela*, *Il1b* and *Tnf* in the mouse liver. METH had no significant effect on the expression levels of the above mRNAs across the groups. (F) Western blotting analysis of the expression of MyD88, TRAF6, p65, IL-1β, and TNF-α in mouse liver. $^{**}p < 0.01$.

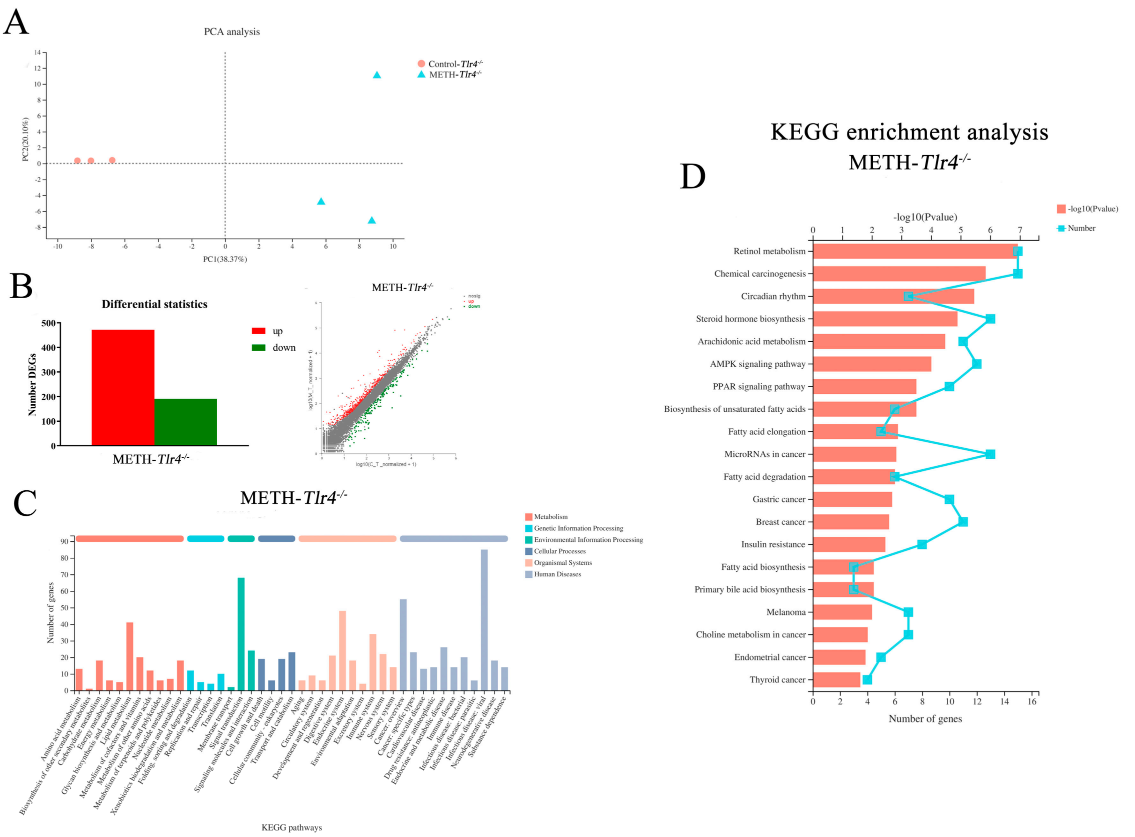


**Supplementary Figure S2** The results of RNA-Seq analysis in *Tlr4*$^{-/-}$ mice. (A) Principal component analysis. (B) Identification of DEGs using the histogram and scatter plot methods (|log2FC| ⩾ 1, p-value < 0.05). (C) KEGG functional annotation analysis for metabolic pathways. (D) KEGG enrichment analysis in METH-treated *Tlr4*$^{-/-}$ mice.

Supplementary Table S1. Top 10 up- and down-regulated genes in METH-treated wild-type (WT) mice compared to the control group

| | Gene name | Regulate | FC (M_WT/C_WT) | *P* adjust |
|---|---|---|---|---|
| 1 | *Gm45140* | up | 603.3 | 5.42E-12 |
| 2 | *E130208F15Rik* | up | 542.1 | 4.04E-11 |
| 3 | *Gm6741* | up | 171.1 | 3.14E-06 |
| 4 | *Gm15429* | up | 163.8 | 1.67E-06 |
| 5 | *Gm28177* | up | 139.9 | 2.54E-13 |
| 6 | *Gm18537* | up | 124.5 | 1.12E-05 |
| 7 | *Zfp91* | up | 113.6 | 0.0355 |
| 8 | *Chrna4* | up | 91.8 | 4.50E-12 |
| 9 | *Slc4a1* | up | 89.0 | 0.0007 |
| 10 | *Gm10131* | up | 83.5 | 0.0003 |
| 11 | *Hsd3b1* | down | -4313.3 | 3.23E-21 |
| 12 | *Gm15832* | down | -1000.0 | 7.36E-06 |
| 13 | *Gm47025* | down | -333.3 | 3.79E-09 |
| 14 | *Gm44775* | down | -166.7 | 9.65E-06 |
| 15 | *Gm20708* | down | -142.9 | 0.0003 |
| 16 | *C330002G04Rik* | down | -111.1 | 6.55E-08 |
| 17 | *Gm7666* | down | -111.1 | 0.0010 |
| 18 | *Gm11537* | down | -100.0 | 0.0004 |
| 19 | *Rps2-ps13* | down | -83.3 | 0.0006 |
| 20 | *Gm36995* | down | -83.3 | 0.0006 |

FC, fold change

Supplementary Table S2. Top 10 up- and down-regulated genes in METH-treated *Tlr4*$^{-/-}$ mice compared to METH-treated wild-type (WT) mice

| | Gene name | Regulate | FC (M_*Tlr4*$^{-/-}$/M_WT) | *P* adjust |
|---|---|---|---|---|
| 1 | *Gm28047* | up | 553.3 | 3.57E-11 |
| 2 | *Ptprn2* | up | 282.1 | 2.54E-08 |
| 3 | *Bsn* | up | 244.0 | 9.90E-23 |
| 4 | *Gm18999* | up | 166.5 | 2.64E-06 |
| 5 | *4930441H08Rik* | up | 139.3 | 1.13E-05 |
| 6 | *Moxd1* | up | 136.0 | 3.87E-08 |
| 7 | *Gm5898* | up | 127.6 | 2.69E-08 |
| 8 | *Htr1d* | up | 79.7 | 0.0007 |
| 9 | *Gm50139* | up | 61.4 | 0.0062 |
| 10 | *Gm18853* | up | 54.1 | 0.0126 |
| 11 | *Gm16867* | down | -1000.0 | 4.47E-12 |
| 12 | *Btnl9* | down | -142.9 | 2.91E-06 |
| 13 | *Cyp2a4* | down | -111.1 | 2.11E-30 |
| 14 | *Gm3776* | down | -100.0 | 0.0005 |
| 15 | *Ccdc69* | down | -90.9 | 5.65E-05 |
| 16 | *Gm11962* | down | -76.9 | 0.0010 |
| 17 | *Gm27177* | down | -71.4 | 5.91E-11 |
| 18 | *Cela2a* | down | -71.4 | 8.09E-06 |
| 19 | *Tlr4* | down | -66.7 | 0.0022 |
| 20 | *Lncbate1* | down | -66.7 | 0.0031 |

FC, fold change

Supplementary Table S3. The DEGs enriched in the Inflammatory mediator regulation of TRP channels and Toll-like receptor signaling pathway in METH-treated *Tlr4*$^{-/-}$ mice compared to METH-treated wild-type (WT) mice

| | Gene name | Regulate | FC (M_*Tlr4*$^{-/-}$/M_WT) | *P* adjust |
|---|---|---|---|---|
| Inflammatory mediator regulation of TRP channels | *Cyp2c55* | down | -3.2 | 2.19E-05 |
| | *Camk2b* | down | -5.2 | 3.74E-19 |
| | *Cyp4a10* | down | -3.6 | 6.16E-05 |
| | *Cyp2c69* | down | -24.5 | 8.20E-06 |
| | *Cyp2c38* | down | -4.4 | 4.14E-19 |
| | *Cyp4a14* | down | -9.1 | 9.90E-23 |
| | *Cyp4a31* | down | -9.3 | 2.68E-17 |
| | *Cyp2c40* | down | -9.5 | 1.56E-23 |
| Toll-like receptor signaling pathway | *Tlr4* | down | -66.7 | 0.0022 |
| | *Tlr12* | down | -15.9 | 2.18E-19 |
| | *Tlr5* | down | -1.9 | 0.0719 |

FC, fold change
